# Supplementary material for: The interactome of intact mitochondria by cross-linking mass spectrometry provides evidence for coexisting respiratory supercomplexes
Source: Mol Cell Proteomics. 2017 Dec 8;17(2):216–32. doi: 10.1074/mcp.RA117.000470 (PMC5795388; doi:10.1074/mcp.RA117.000470)
Supplement: Supplemental Data [file supp_RA117.000470_133922_0_supp_23978_fzffwf.zip › spectra_annotation/mito_spectra_annotation/304-1-5-1-10-1.pdf]

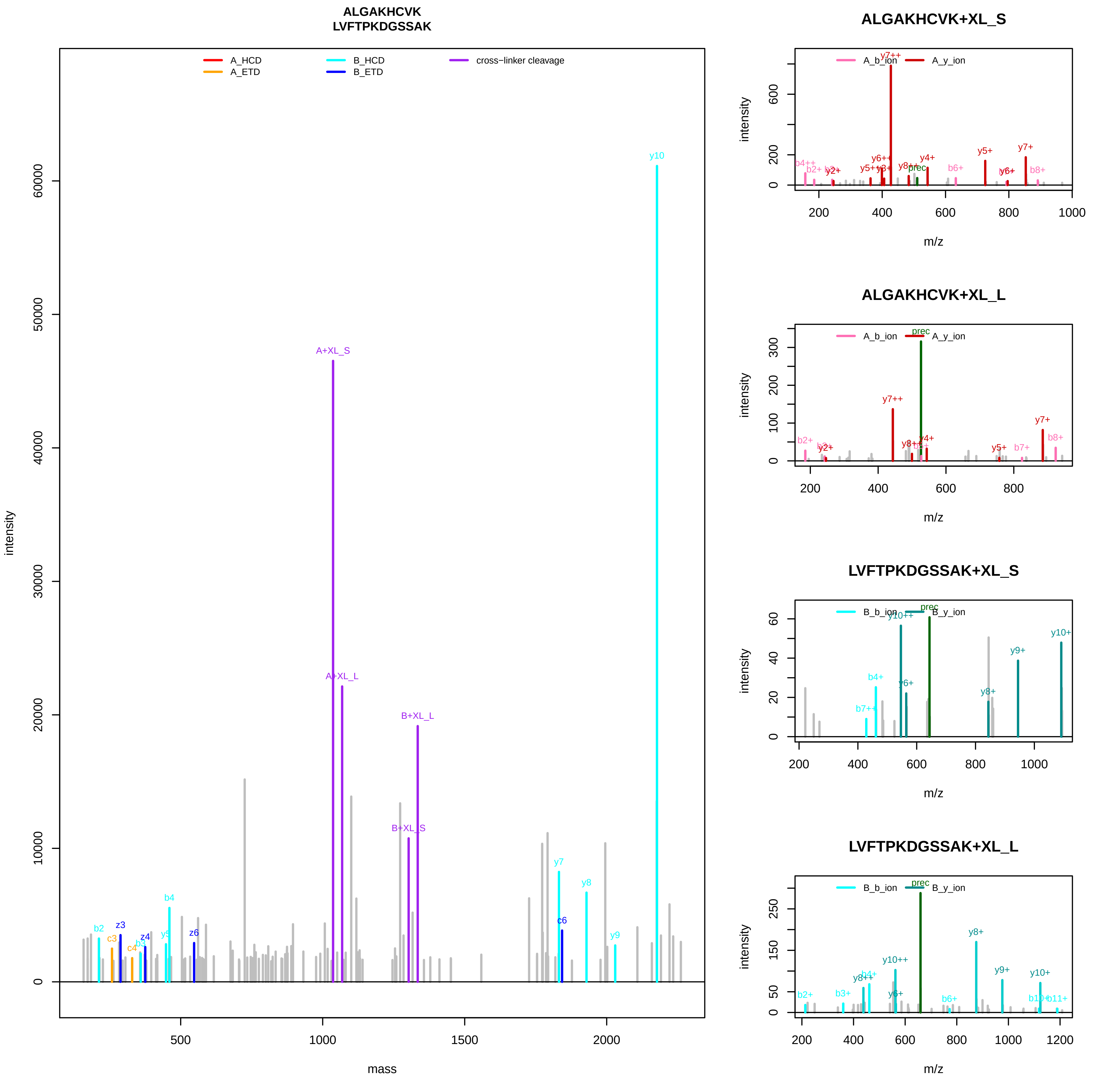
ALGAKHCVK
LVFTPKDGSSAK
A_HCD
A_ETD
B_HCD
B_ETD
cross−linker cleavage
intensity
mass
0
10000
20000
30000
40000
50000
60000
500
1000
1500
2000
A+XL_S
A+XL_L
B+XL_L
B+XL_S
y10
y7
y8
y9
c6
b2
z3
c3
c4
b3
z4
y5
b4
z6
ALGAKHCVK+XL_S
ALGAKHCVK+XL_L
LVFTPKDGSSAK+XL_S
LVFTPKDGSSAK+XL_L
A_b_ion
A_y_ion
B_b_ion
B_y_ion
prec
m/z
y7++
y7+
y5+
y4+
y6++
y8++
y2+
b6+
b8+
b2+
b7+
y10++
y10+
y9+
y8+
y6+
b4+
b7++
b3+
b6+
y8++
b4+
b10+
b11+
